# Bone mineral density improvement after 48 weeks of switch to maraviroc+darunavir/ritonavir 300/800/100 mg QD, preliminary results of GUSTA study

**DOI:** 10.7448/IAS.17.4.19816

**Published:** 2014-11-02

**Authors:** Claudia Bianco, Barbara Rossetti, Roberta Gagliardini, Silvia Lamonica, Luri Fanti, Francesca Lombardi, Roberto Cauda, Simona Di Giambenedetto, Andrea De Luca

**Affiliations:** 1 Infectious Diseases Unit, Azienda Ospedaliera Universitaria Senese, Siena, Italy; 2 Clinic of Infectious Diseases, Catholic University of Sacred Heart, Roma, Italy

## Abstract

**Introduction:**

Low bone mineral density (BMD) and osteoporosis are prevalent in HIV-infected patients and were associated with HIV infection and tenofovir-containing ART.

**Materials and Methods:**

The GUSTA study (GUided Simplification with Tropism Assay) is a two-arm, prospective, multicenter, 1:1 randomized controlled trial designed to demonstrate the non-inferiority of therapeutic switch to maraviroc+darunavir/ritonavir (MVC+DRV/r) 300/800/100 mg QD against the continuation of previous triple cART in patients with stable virological suppression. Enrolment criteria include HIV1-RNA <50 copies/mL for >6 months, R5 tropism and CD4>200 cells/µL for >3 months. Dual energy X-ray absorptiometry scans of proximal femur and lumbar spine were performed at baseline and week 48. Bone composition was evaluated using L2-L4 lumbar column and proximal femoral BMD, T-score and the Z-score. At the same timepoints, plasma bone metabolism biomarkers were measured. Linear regression was used to compare means of differences between arms. The association between BMD changes and the baseline variables was assessed by linear regression.

**Results:**

27 patients were included, 13 from study group and 14 from control group, 74.1% were males, 44.4% heterosexuals, 81.5% Caucasian, median age was 47 years (IQR 41–53), time from HIV diagnosis 13.4 years (9–19), CD4 553/µL (406–739), nadir CD4 201/µL (76–283). At baseline, median ART duration was 10.5 years (5.7–15.3), the majority of patients (70.4%) was on tenofovir, 63% was on a PI-based regimen and 14,8% on an NNRTI-based regimen. Mean proximal femur BMD from baseline increased over 48 weeks by 2.06% (SD 2.24) in the study arm and decreased by −2.77% (SD 4.63) in control arm (p=0.003). The change over 48 weeks in proximal femur T-score was significantly different between the study (+0.11, SD 0.22) and control arm (−1.14, SD 0.27, p=0.016). Also the changes in total alkalin phosphatase (−20 U/L vs −1.5, p=0.003) was significant between the two groups. After adjusting for time from HIV diagnosis and years of ART, study group was the only factor associated to higher mean percentage change from baseline femoral BMD (MVC+DRV/r +4.83, p=0.044).

**Conclusions:**

The study demonstrated a significant improvement in femoral BMD and T-score after treatment simplification with MVC+DRV/r.

